# Analyzing Passive BCI Signals to Control Adaptive Automation Devices

**DOI:** 10.3390/s19143042

**Published:** 2019-07-10

**Authors:** Ghada Al-Hudhud, Layla Alqahtani, Heyam Albaity, Duaa Alsaeed, Isra Al-Turaiki

**Affiliations:** 1 Information Technology Department, College of Computer and Information Sciences, King Saud University, Riyadh 12371, Saudi Arabia; 2 King Abdulaziz City for Science and Technology, National Satellite Technology Center, Riyadh 12354, Saudi Arabia

**Keywords:** Passive Brain Signals, adaptive automation and controller, EOG artifact, independent component analysis, engagement index

## Abstract

Brain computer interfaces are currently considered to greatly enhance assistive technologies and improve the experiences of people with special needs in the workplace. The proposed adaptive control model for smart offices provides a complete prototype that senses an environment’s temperature and lighting and responds to users’ feelings in terms of their comfort and engagement levels. The model comprises the following components: (a) sensors to sense the environment, including temperature and brightness sensors, and a headset that collects *electroencephalogram* (EEG) signals, which represent workers’ comfort levels; (b) an application that analyzes workers’ feelings regarding their willingness to adjust to a space based on an analysis of collected data and that determines workers’ attention levels and, thus, engagement; and (c) actuators to adjust the temperature and/or lighting. This research implemented independent component analysis to remove eye movement artifacts from the EEG signals and used an engagement index to calculate engagement levels. This research is expected to add value to research on smart city infrastructures and on assistive technologies to increase productivity in smart offices.

## 1. Introduction

Worker engagement and concentration are essential to ensure productivity in the workplace. However, busy workers may find it hard to concentrate since their focus can be easily broken by many factors, and this may affect their engagement at work. The environment surrounding the employee is one of these factors [1]. A room’s temperature [2], brightness level, window size [3], and noise level [4] can affect focus at work, especially when employees have a busy schedule. For instance, small changes in room temperature may directly affect engagement, which influences the productivity of employees; this may sometimes occur without anyone noticing the causes for it. Therefore, providing a control system to maintain an environment that helps increase user engagement can improve productivity at work. Furthermore, the control system’s level of interaction with users in maintaining an appropriate environment is critical, as most busy workers find it difficult or time consuming to track their environment in order to continually adjust it. Although such a control system sounds promising as an assistive technology to accommodate workers with movement disabilities, it would be impractical in offices with more than one worker. Hence, the proposed research assumed the environment of a small office with one or two workers that is equipped with sufficient infrastructure to assist workers with disabilities.

### 1.1. Passive Brain Computer Interaction

The original goal of *Brain Computer Interaction* (BCI) is to provide a communication and a control channel for people with severe disabilities, especially those who are completely paralyzed. Most BCIs are used for direct or *explicit* control, which might involve users controlling a cursor or selecting letters on a computer screen using mental activity. The channel transfer rate of these applications remains under 25 bits per minute [5] Such explicit BCIs often require a long training period but remain a solution for patients.

*Non-command* user interfaces [6] have been proposed to use a BCI as an implicit communication channel between a user and a computer. Implicit or passive BCIs refer to BCIs in which the user does not try to control their brain activity. Passive BCIs have been deployed in recent research on adaptive automation. In the field of adaptive automation, the first brain-based system was developed by Pope et al. [7]. In this system, tracking tasks were allocated between a human and a machine based on an engagement index, which was calculated using users’ brain activity. More recently, Kohlmorgen et al. presented the use of implicit BCI in the context of a real driving environment [8]. In this study, the user was engaged in a task mimicking an interaction with the vehicle’s electronic warning and information systems. This task was interrupted when a high mental workload was detected. This experiment showed good reaction times on average using BCI based on implicit interaction.

### 1.2. BCIs in the Office

The environment around a person working in an office has a direct effect on their engagement and productivity [2,4]. In the last few years, BCI researchers have studied BCI technology and its uses, both for disabled and healthy users [9]. Finding an easy and smart way to automatically detect the best environmental conditions and then adjust them accordingly would make the work environment a more enjoyable and proactive place. Recent research has been conducted to develop smart offices using various technologies and techniques, such as smartphones [10], speech commands, gestures [11], and even active brain signals [12]. While some systems do not require direct interaction with the user, most do. Such an interaction involves using passive BCI signals and concentrating on only one environmental factor, such as a window [13]. However, to our knowledge, no research has been conducted that includes adjusting multiple factors in the office environment to enhance worker engagement and concentration [13]. Thus, an intelligent system that passively observes workers’ mental status, automatically and passively acquires their brain signals, analyzes these signals alongside environmental measurements to find the perfect state to enhance workers’ concentration, and adjusts environmental factors to meet the required state is needed.

## 2. Background and Literature Review

Using BCIs as a tool in smart offices [12] and homes [14,15] varies in terms of purpose, methodology, and environment. Hence, in this section, a background on brain wave types and the use of BCI technologies and methodologies in smart offices is discussed.

### 2.1. Brainwaves

Brain waves are classified based on bandwidth, and each type serves a different function. Low-frequency waves dominate when a person is tired or daydreaming. High-frequency waves appear more often when a person is active. The bandwidths of brain waves are shown in Table 1.

### 2.2. BCI

**Figure 1:**
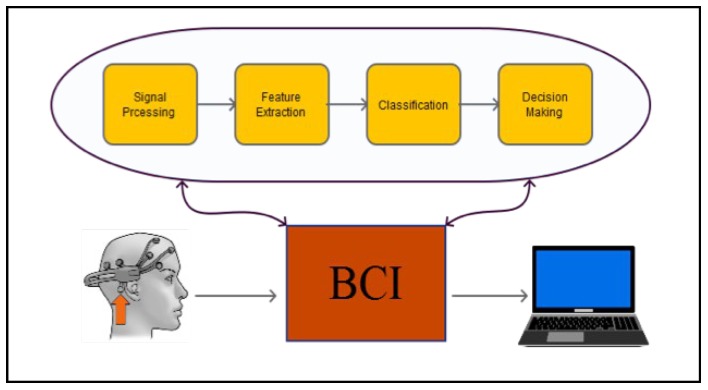
Typical Brain Computer Interaction structure, including data acquisition and signal processing; finally, the interpreted action is shown as a result.

*Electroencephalogram* (EEG) electrodes can be used to measure the voltage resulting from brain activities. In the signal-processing stage, several steps are taken to obtain control signal, including preprocessing, feature extraction, and classification. In the last stage, the processed signal is interpreted into the desired action. Figure 1 describes these stages.

**Figure 2:**
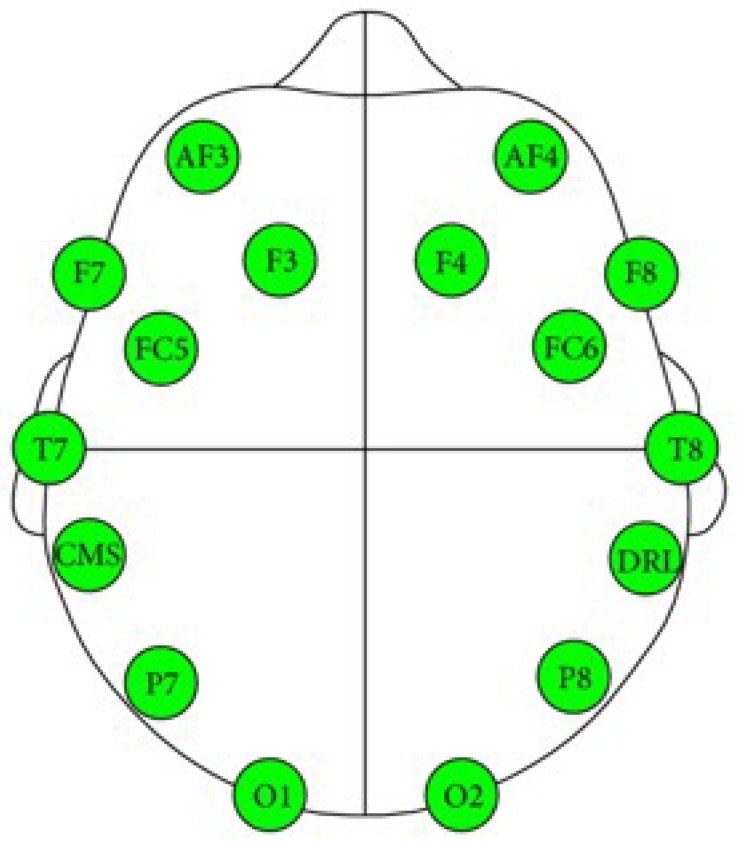
Emotiv EEG neuroheadset sensor position [18].

Advancements in the development of BCI systems in recent years have helped to make them more appealing to a wider range of user groups. The cost of such systems has dramatically dropped, and they have become more convenient to use; the electrodes are now wireless, dry, and easy to move during wear. Currently, many commercial BCI devices are available, including NeuroSky [16] and EMOTIV EPOC [17]. EMOTIV EPOC is a BCI device that was developed for research and development applications. It contains 14 sensors to acquire brain signals at the following locations: AF3, F7, F3, FC5, T7, P7, O1, O2, P8, T8, FC6, F4, F8, and AF4, as shown in Figure 2 [18].

BCIs vary in terms of their properties and the ways they acquire, analyze, and translate signals. Typical BCIs involve brain signal acquisition, processing, and interpretation. Brain signals can be acquired using various methods, such as EEG.

### 2.3. Electrooculography/Electromyogram Artifact Removal

Artifacts are the undesirable signals and noise that can interfere with acquired brain signals. Artifacts have a much stronger amplitude than EEG signals and may affect the acquired brain signals, thereby reducing the performance of BCIs. There are two types of artifacts: physiological and non-physiological. Common physiological artifacts include eye movements, which are detected by *electrooculography*(EOG), and muscle movements, which are detected by *electromyography* (EMG). They usually appear as large-amplitude, high-frequency distortions within brain signals. Non-physiological artifacts are usually technical and caused by the environment; they include power-line noises and disturbances caused by recording equipment (e.g., changes in electrode impedances). Non-physiological artifacts are easy to handle and prevent (by applying filtering and the proper recording procedure, respectively). However, physiological artifacts are challenging to eliminate from brain signals and are a significant problem in designing BCIs [19]. Recently, researchers have published many methods to remove eye movement and blinking artifacts from EEG data. Among these methods is rejecting contaminated EEG epochs; however, this method results in a significant loss of collected information. Another method is performing regression on simultaneous EEG recordings, including EMG and EOG recordings in the time or frequency domain. This method aims to derive the parameters that characterize the appearance and spread of EOG artifacts in EEG channels. EOG records may also contain brain signals; hence, removing EOG activity would result in the loss of relevant EEG signals. As there is no clear reference channel for the artifacts, regression methods cannot be used to removed them. A recent method was proposed by Hsu et al. 2016 [20] that involves applying *Independent Component Analysis* (ICA) to eliminate artifacts from EEG sensors. In comparison with results obtained using regression-based methods and principal component analysis, Hsu et al.’s published results show that ICA can effectively detect, separate, and remove artifacts from EEG records.

### 2.4. Processing EEG to Measure Engagement Levels

The growth of the BCI technology has attracted many researchers, who often use this technology to measure the engagement of a user. The purpose of measuring user engagement differs from that of enhancing user experience, interfaces, games, and online learning systems. For example, [21] developed an attention-aware system that monitors a student’s attention to an online education system and alerts their teacher when attention decreases. The system uses NeuroSky and achieved an 89.52% accuracy rate on average. Ref. [22] developed a prototype system to enhance user experience in museums. This system uses real-time feedback regarding user engagement, using a BCI to provide a tailored museum experience based on a user’s taste. The system provides guided tours and suggests exhibits based on a user’s engagement level.

A smart office was presented for the first time in [11], in which a user was observed and their intentions anticipated to augment their environment and communicate useful information. At first, the system was controlled using voice commands and gestures [11]. In recent years, much research has adapted BCIs for use in smart offices in order to enhance worker experience and productivity. The system in [13] was designed to improve user engagement by blocking outside distractions; this was done by controlling the opacity of an office’s glass wall. The system uses BCI to passively measure a user’s level of engagement through NeuroSky’s ThinkGear device. The user’s level of engagement was used to determine the opacity of an electrochromic smart glass tile, which could change from being fully opaque to being fully transparent. As the user focused, the system increased the opacity of the window as a signal for others to not to disturb the user. However, this system ignores other surrounding factors, such as lighting and room temperature. Ref. [12] used an EMOTIV headset to actively acquire a worker’s brain signals and translate them to control the office environment. The system allows users to control the temperature and brightness using their thoughts. However, the system does not control the environment passively; it requires user intervention. Therefore, using BCIs to develop smart offices is a growing research area that still has room for improvement.

## 3. Methodology

### 3.1. Proposed Structure

**Figure 3:**
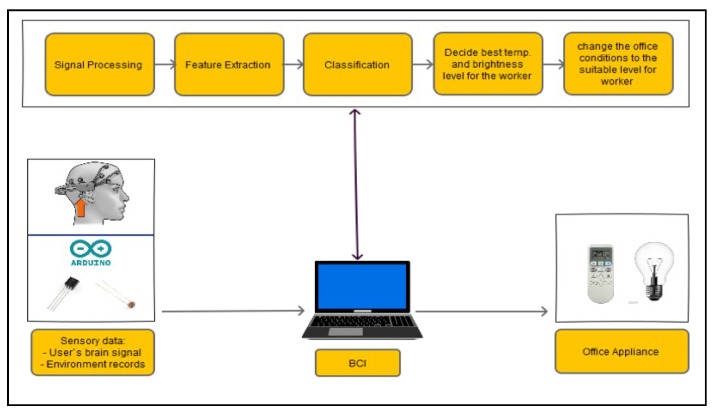
Basic structure of the proposed smart office controller.

The basic structure of the proposed system is divided into the following phases: brain and environment signal acquisition, signal processing, user engagement calculation, and decision making, as shown in Figure 3. These phases continue working in a cycle to ensure the continuing functionality of the system in order to provide a suitable environment for the worker.

### 3.2. Development Environments and Tools

LabVIEW (also called G) is a dataflow programming language that uses virtual instruments to represent a program. In the field of BCI research, the system in [23,24] used LabVIEW as the development environment to build a system for a smart house. The system’s purpose is to monitor the temperature, humidity, lighting, fire and burglar alarms, and gas density in the house in order to ensure safety. Ref. [25] built a smart home system based on a wireless sensor network designed to ensure the safety of elderly people living alone. However, neither of these studies used BCIs. Although many researchers have used LabVIEW to develop BCIs, none were used to create smart offices.

### 3.3. Signal Acquisition

The proposed system controls the office environment automatically as it detects the user’s engagement and intensity levels and the temperature of the environment, which it then maps onto the user’s comfort level. Hence, the system adjusts temperature and light intensity as needed. An EMOTIV headset is placed on a user’s scalp to collect real-time data (brain signals) in various situations (comforted, stressed, engaged, and distracted), while environmental sensors acquire the temperature and brightness level. The sampling frequency of the headset is fixed at 128 samples per second. Since electrodes placed on the frontal and occipital lobes perform better in obtaining cognitive EEG data than in other locations, the electrodes are placed at the following locations: F3, F4, FC5, FC6, P7, P8, O1, and O2. These channels were chosen because they are closest to those used in other engagement research [22,26,27,28].

To acquire environmental signals, a temperature sensor was used, and a photoresistor was used to acquire the light intensity. The sensors were set on an electronic circuit connected to an Arduino UNO board, which functions as an interface between the sensors and the computer.

### 3.4. Signal Processing

After the brain and environmental signals are acquired, they are imported for processing. The processing of the temperature and light signals is done by conditioning them. The temperature and light signals are analog signals; signal conditioning involves converting these signals into digital signals for the next stage. This conversion is done using an existing function in LabVIEW.

**Figure 4:**
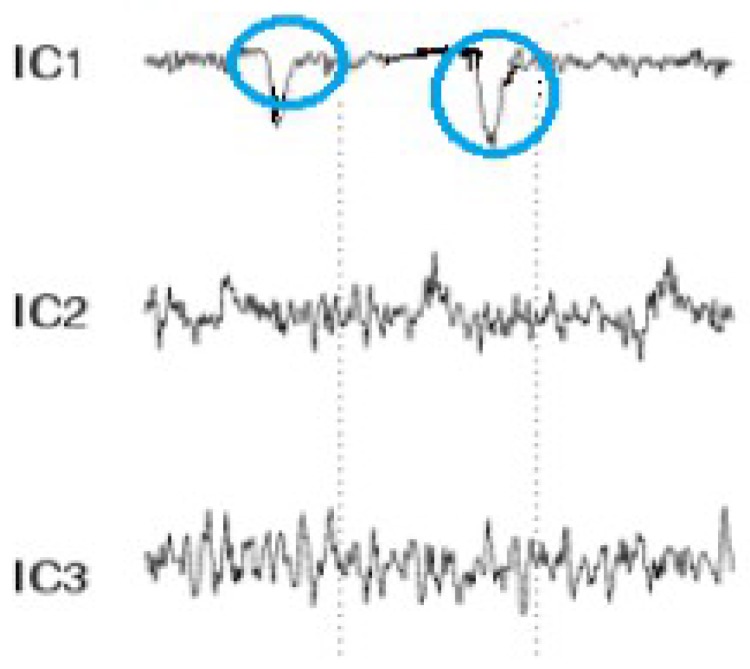
Mixed electroencephalogram/electrooculography (EEG/EOG) data. Note the pulses in the independent components.

**Figure 5:**
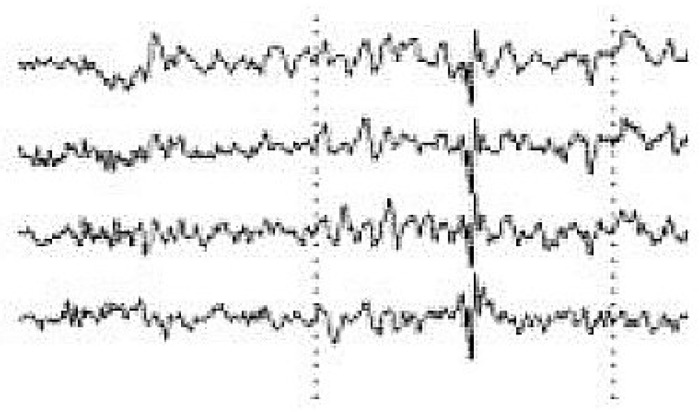
EEG data after eye blink removal.

The signal processing of the acquired EEG signals is considered difficult due to noise and artifacts. As such, this process is divided into the following stages in order to extract the frequencies required for the task:

- A high-pass filter with a cut-off frequency of 0.6 Hz and a low-pass filter with a cutoff frequency of 50 Hz are used to remove the DC offset.
- Linear finite impulse response filtering is used to remove 50- or 60-Hz line noise.
- Since the reading is collected in real time, a buffer is used to read and then remove the mean of n samples.
- A band-pass filter is used to remove frequencies that are not related to the task. Since only alpha, beta, and theta waves are required, frequencies outside the ranges of 13–22 Hz, 8–12 Hz, and 5–7 Hz are eliminated. An *Infinite Impulse Response* filter of the second order is used as a band-pass filter to filter alpha and beta waves.
- EOG artifacts represent the comfort level of a worker trying to relax by closing the eyes. Such artifacts include eye movements. Blinks are separated using ICA in order to tangibly improve the EEG data interpretation and analysis. ICA is used to identify, separate, and remove these artifacts with a minimal loss of brain activity data. The algorithm follows the steps described below:
  1. Figure 4 shows all the components, including those that contain eye artifacts and spatial mixtures of brain and artifact activities.
  2. The rows of the input matrix, X, are EEG signals recorded with different electrodes, and the columns are measurements recorded at different time points, where participants were instructed regarding their eye movement. Hence, the input data are weighted as follows: C = MX.
  3. ICA-based artifact separation from EEG data is performed using linear decomposition. The inverse matrix of M represents the projection strength of the components at each of the brain sensors. After linear separation, the multi-channel data result is a sum of independent and spatially fixed components.
  4. Components that represent artifacts based on spatial filters are extracted.
  5. Artifactual components, including blink artifacts, are removed. Eye movement artifacts are isolated as a linear subtraction of the components representing the artifacts, as shown in Figure 5.

### 3.5. Engagement Index Computations and Environmental Control

This system uses the engagement value to determine the best action. If a new high engagement value is recorded, the system saves its related temperature and light intensity values to use when controlling the system. Since temperature and light intensity do not change suddenly in smart offices, the overload on the system is reduced, making it more efficient. The system logs all environmental data and their related engagement levels as references to find the best condition for the user.

- Monitor the surrounding office environment (room/body temperature, brightness level, curtain state) using special sensors.
- Analyze these data to predict the best possible environment to help the user stay focused.
- Associate the brain signal data with the conditions in the second step above.
- If the statement (current engagement score > maximum engagement) is true, then the system will first set a new maximum engagement value and then acquire the temperature and light intensity using the acquisition function. The system only acquires the temperature and light intensity here.
- If the statement (current engagement score > maximum engagement) is false, the system has detected a low engagement level and will find the most suitable environment to raise engagement; then, it adjusts the office environment to this environment. First, the system checks whether current engagement is low; if so, then it sets the temperature and light intensity values to the saved ones. After, the system logs the changes.
- If the engagement value is normal, the system does nothing.
- The system automatically adjusts the office environment to maintain a user’s engagement when it is high.

### 3.6. Feature Selection and Classification of Actions

**Figure 6:**
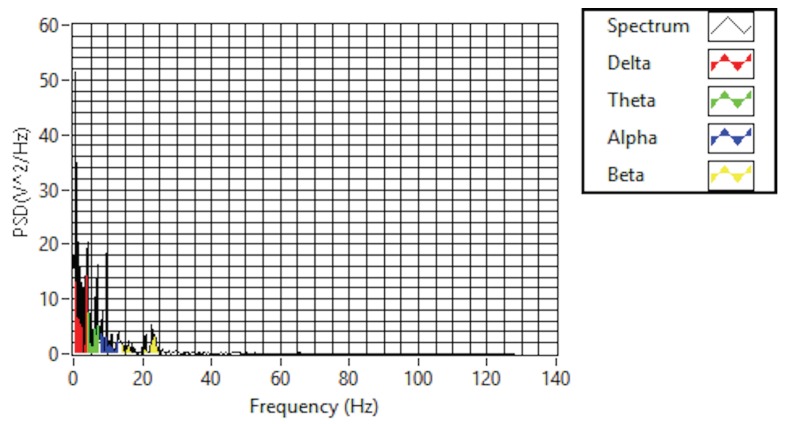
EEG data spectrum.

The purpose of feature selection is to convert digitized brain signals that are recorded at various locations into features [20]. For the feature extraction, a fast Fourier transform (FFT) is used. A Hanning window is first applied; then, the FFT is performed for each epoch. After, the average power spectral density value is extracted for the alpha, beta, and theta frequency bands in order to extract the features of each band. The FFT reduces the computation complexity by obtaining faster results than the discrete Fourier transform. In addition, the FFT is a commonly used algorithm for signal processing; in this project, it is used to capture the frequency components of the EEG signals recorded by the EMOTIV headset. Figure 6 shows a real-time EEG data spectrum. The system acquires new EEG data and processes these data in each iteration. Then, it identifies the engagement level of a user by analyzing the extracted features associated with their mental status using a suitable classification. The alpha, beta, and theta powers collected previously are used to calculate the engagement score. The engagement index is calculated using the following function:

(1) $$\mathrm{Engagement}\;\mathrm{level}=\frac{\beta }{\alpha +\theta }$$

Then, the engagement values are scaled from zero to one; the higher the value is, the higher engagement level is. In this stage, the system makes a decision and applies changes depending on the results of the previous stage. All the environmental data and engagement data are logged. In a case where the engagement level is below the threshold, the system takes action. First, the system checks whether the environmental data are in the normal range to ensure that the cause of this loss is the environment. If so, the system adjusts the environmental data to a suitable range (from the log data) and then notifies the user about the changes. The decision algorithm is shown in Algorithm 1.

**Algorithm 1** Decision algorithm.**if** engagement value ≤ Threshold **then**
1:Find temperature and light intensity values associated with Max Eng. Value.2:Adjust the temperature to it;3:Adjust the light intensity to it;4:Notify the user about changes; **else** Do Nothing **end if**=0


## 4. Experimental Setup

The raw EEG data were collected from two participants, both male and aged 20–29 years of age. The mental status and health conditions of both participants were normal. For this experiment, there was no pre-knowledge required from the participant about how to use a computer or how to operate an office appliance. The experiment took place in a room where the participants were asked to perform specific tasks that required their attention. The tasks included sending emails, editing a Word document, and reading a document. The participants performed the tasks wearing a headset connected to a computer containing the system software. The headset sampling frequency was set to 256 samples per second. In addition, an Arduino board with two circuits containing a temperature sensor and light sensor were connected to the same computer.

## 5. Discussion

In this section, the results are presented in more detail and analyzed in three phases. The first phase records the values of a user’s engagement level at a neutral status in order to learn and record the engagement level of the user. The second phase analyzes the impact of changing the temperature and fixing the light intensity value on the user’s engagement level. The third phase analyzes the effect of changing the light intensity value while fixing the temperature on the user’s engagement level.

**Figure 7:**
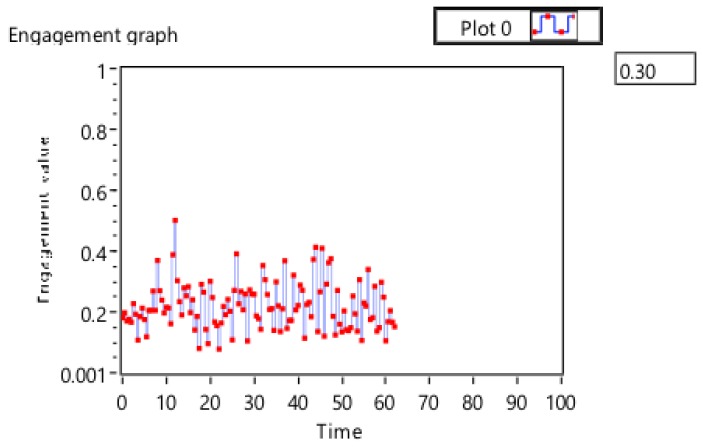
Engagement data for one session.

**Figure 8:**
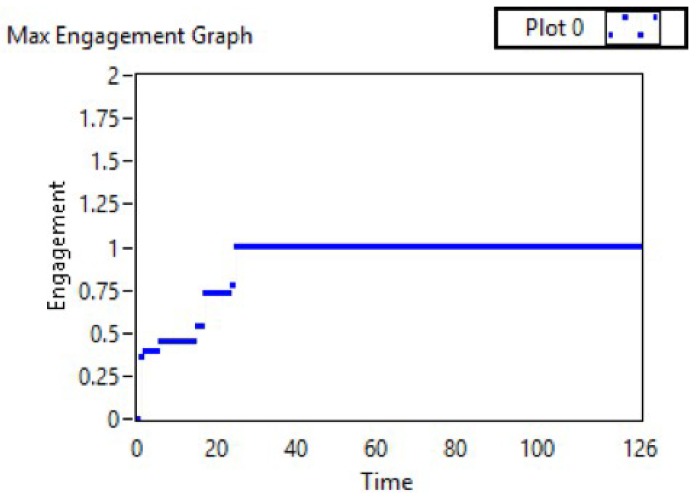
Engagement and sensor results for one session.

Figure 7 shows the engagement across one full session. Figure 8 presents the maximum engagement, temperature, and light intensity values over time to show changes to the system over time and to allow for a rapid analysis of the system’s performance and the user’s engagement.

Table 2 shows the results of calculating the maximum engagement value and its related environment sensory readings. The system accurately saved the high engagement value. The highest score in this session was achieved at a temperature of 24 and a light intensity of 86. By observing the engagement recorded after changing the office environment status, an improvement in engagement was seen for a reasonable time (Table 2). The recording, at first, was below the threshold (0.342805); after setting the temperature to 24.9 and the light intensity to 86.14, the engagement score slightly increased.

### 5.1. Maximum Engagement Records and Associated Temperature and Light Intensity

**Figure 9:**
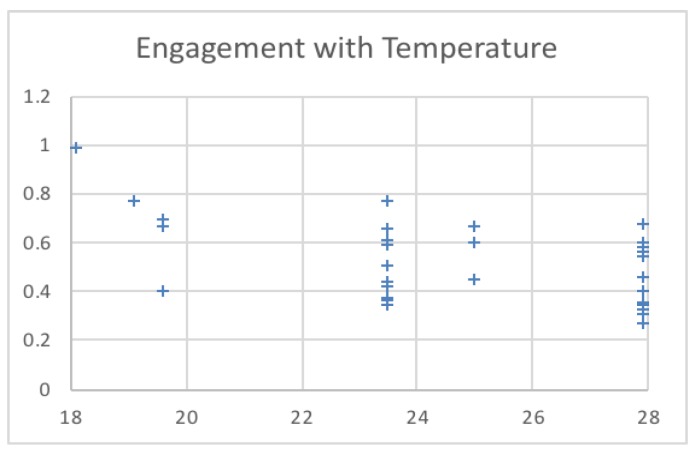
Engagement values for different temperatures.

**Figure 10:**
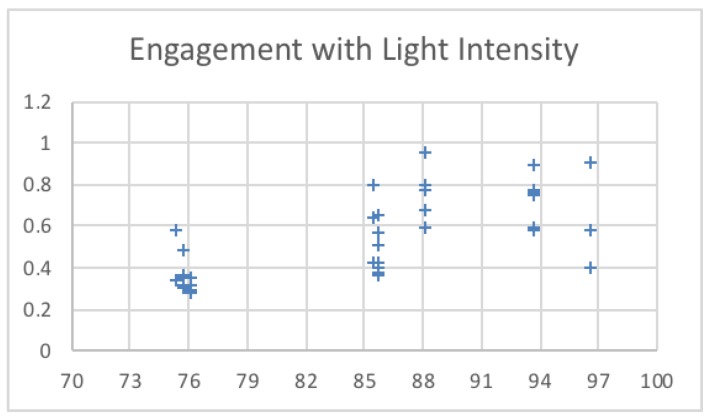
Engagement values for different light intensity values.

Table 3 shows the temperature and light intensity values related to the maximum engagement value from each session. The engagement values changed over 15 min across the three sessions. The first session was run under a low temperature (18–20), as shown in Figure 9. The second session was run under a high temperature (24–26), and the third session was run in the middle of the low and high temperatures used (21–23). The system calculated the engagement values and saved the maximum engagement values. The light intensity remained in the same range. Figure 10 shows the changes to the saved maximum engagement values over 15 min for each session.

### 5.2. Engagement versus Synthesized Changing Temperature Values

In this phase, the temperature values were changed to record the associated engagement values. The results show the influence of changes in temperature on the engagement level of the user; our results conform to those reported in [7,29].

**Figure 11:**
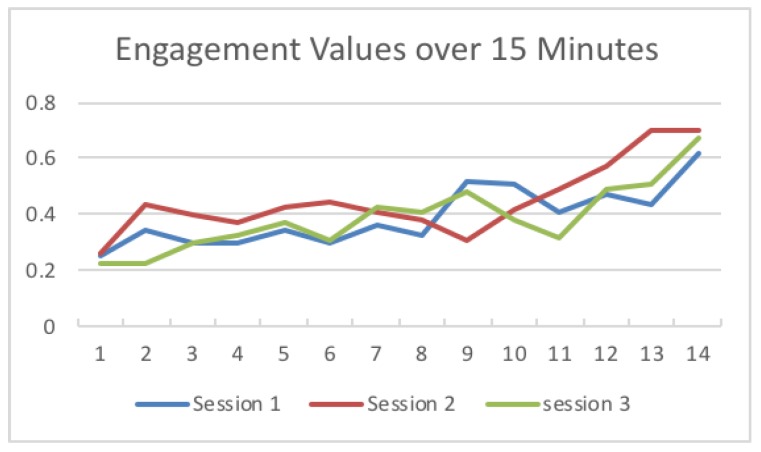
Engagement values over 15 min.

By changing the temperature and light intensity (Figure 9 and Figure 10), at first, all sessions started with low engagement values, at 0.25056, 0.266476, and 0.225049. Since these values are below the threshold (0.4), the system set the temperature and light intensity values to the saved optimal values for each session. After, as shown in Figure 11, the engagement values slightly increased over time until they reached high values (above 0.6) at the end of the sessions.

## 6. Conclusions

EEG is expected to be a future user input technology. This research provides a prototype and a first step to relate EEG data with the office environment in order to enhance and develop smart offices. The environmental sensory data remained the same from sensors connected to Arduino board. Alpha, beta, and theta powers were extracted from the EEG data and used to calculate user engagement. Based on the engagement value, the system sets the temperature and light density values. The system saved 22.3 C as the optimal temperature and 76 lux as the optimal light intensity.

The experimental results show efficient control in terms of the focus level of users by correctly adjusting the office temperature and light intensity.

In this study, the best possible environment was determined based on the engagement value. There are two factors considered in this study: room temperature and illumination. The value for each was set based on the highest engagement value obtained. In the future, Artificial Intelligence algorithms may be utilized to determine the best environment. The temperature and lumen values in this study are assumed to be fixed. However, during the testing phase we changed the values within a limited range (range of normal situation in offices).

Many factors affect EEG, including: emotional state, fatigue, sleepiness, age, body temperature, and blood oxygen saturation. All these factors are important to consider. Thus, it is suggested to collect more feedback from the subjects and also to vary the duration and frequency of training sessions.

Due to time and device limitations, our system only deals with room temperature and lighting. Future enhancements should count more than two parameters and investigate the use of other mechanisms to track eye blinking as a sign of discomfort. In addition, low engagement should be eliminated using linear discriminant analysis for the classification of the feature vectors extracted from the ICA components. Further enhancements could use a more convenient EEG headset—this may generate effective results since the user would not feel a difference in their daily routine at the office.

## Figures and Tables

**Table 1:** Brainwave bandwidths and functions.

Name	Speed	Description	Wave Sample
Infra-low	<0.5 HZ	This is the basic cortical tune that underlies higher brain functions. This type of wave is very slow, which makes it hard to detect; therefore, limited knowledge exists surrounding it.	N/A
Delta	0.5 to 3 HZ	This wave is usually associated with deep stages of sleep and meditation. In addition, it has the highest amplitude and the slowest rate.	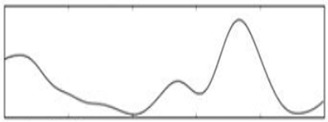
Theta	3 to 8 HZ	This is a low-frequency and low-altitude wave that occurs in sleep, daydreaming, and meditation.	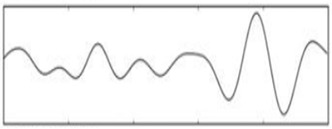
Alpha	8 to 12 HZ	This wave is generated by the occipital lobe when closing the eyes or relaxing. It is most visible over the parietal and occipital lobes.	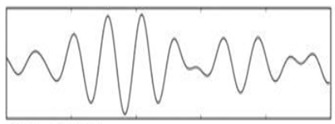
Beta	12 to 38 HZ	This wave dominates most of the human waking state. This wave becomes small and fast when performing hard mental work, such as problem-solving, decision-making, etc. It is most prominent in the frontal cortex during intense and focused mental activity.	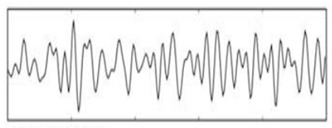
Gamma	38 to 42 HZ	This is the fastest brain wave and occurs when a person is facing a sudden situation.	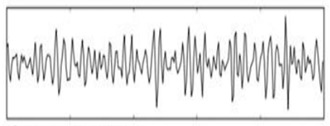

**Table 2:** Calculating the maximum engagement results for one session.

Maximum Engagement Score	Temperature Value	Light Intensity Value
0.225049	25.459999	86.044
0.302148	25.459999	86.142
0.32732	25.459999	85.848
0.369682	25.459999	85.946
0.406814	25.459999	85.946
0.684875	25.459999	85.652
0.936263	24.969999	86.142

**Table 3:** Related temperature and light intensity values.

Session	Temperature	Light Intensity
Session 1	19.000	90.944
Session 2	25.400	75.600
Session 3	23.500	77.028
